# Preventing adverse events of chemotherapy by educating patients about the nocebo effect (RENNO study) – study protocol of a randomized controlled trial with gastrointestinal cancer patients

**DOI:** 10.1186/s12885-018-4814-7

**Published:** 2018-09-24

**Authors:** Julia Quidde, Yiqi Pan, Melanie Salm, Armin Hendi, Sven Nilsson, Karin Oechsle, Alexander Stein, Yvonne Nestoriuc

**Affiliations:** 1 Department of Oncology, Haematology, Bone Marrow Transplantation with Section Pneumology, University Medical Center Hamburg-Eppendorf, Martinistr. 52, 20246 Hamburg, Germany; 2 Department of Psychosomatic Medicine and Psychotherapy, University Medical Center Hamburg-Eppendorf, Martinistr. 52, 20246 Hamburg, Germany; 3 Clinical Psychology and Psychotherapy, University of Hamburg, Von-Melle-Park 5, 20146 Hamburg, Germany; 4 University Cancer Center Hamburg, Hubertus Wald Tumorzentrum, University Hospital Hamburg-Eppendorf, Martinistr. 52, 20246 Hamburg, Germany

**Keywords:** Side effects, Chemotherapy, Gastrointestinal cancer, Treatment expectations, Nocebo effect, Informed consent

## Abstract

**Background:**

Patients undergoing chemotherapy are highly burdened by side effects. These may be caused by the pharmacodynamics of the drug or be driven by psychological factors such as negative expectations or pre-conditioning, which reflect nocebo effects. As such, negative pre-treatment expectations or prior experiences might exacerbate the burden of chemotherapy side effects. Educating patients about this nocebo effect has been put forward as a potential strategy to optimize patients’ pre-treatment expectations. In this study, we evaluate whether a briefing about the nocebo effect is efficacious in reducing side effects.

**Methods:**

In this exploratory study, a total number of *n* = 100 outpatients with newly diagnosed gastrointestinal cancers are randomized 1:1 to an information session about the nocebo effect (nocebo-education) or an attention control group (ACG) with matching interaction time. Assessments take place before the intervention (T1 pre), post-intervention (T1 post), and 10 days (T2) and 12 weeks (T3) after the initial chemotherapy. The primary outcomes are the patient-rated number and intensity of side effects at 10-days and at 12-weeks follow-up. Secondary outcomes include coping with side effects, tendency to misattribute symptoms, compliance intention, attitude towards the chemotherapy, co-medication to treat side effects and the clinician-rated severity of toxicity. Further analyses are conducted to investigate whether a potential beneficial effect is mediated by a change of expectations before and after the intervention.

**Discussion:**

Informing patients about the nocebo effect might be an innovative and feasible intervention to reduce the burden of side effects and strengthen patients’ perceived control over adverse symptoms.

**Trial registration:**

The trial is registered at the German Clinical Trials Register (ID: DRKS00009501; retrospectively registered on March 27, 2018). The first patient was enrolled on September 29, 2015.

## Background

Gastrointestinal tumors, including cancer of the esophagus, stomach, colon, rectum, anus, pancreas, gallbladder and bile duct, constitute the largest group of cancer for men, and the second largest group of cancer for women [1]. Further, they are characterized by high mortality rates [1]. One of the treatment options for gastrointestinal tumors is chemotherapy, which can be indicated in the neoadjuvant, adjuvant or palliative setting [2]. Due to the high toxicity of the chemotherapy, cancer patients are commonly burdened by side effects of varying severity which may impair quality-of-life [3, 4]. Furthermore, side effects have substantial weight in the individual decision process of whether or not to proceed with chemotherapy and were shown to be one of the three major predictors of treatment discontinuation in the palliative setting [5, 6]. Strikingly, side effects are not fully defined by the pharmacodynamics of a given drug, but can also be caused by psychological factors. These so-called nocebo effects are best known as the counterparts of placebo effects [7]. The nocebo effect can depend on a variety of factors, e.g. the patient-practitioner relationship, a patient’s treatment expectations, or prior learning experiences [8]. Nocebo-driven side effects can occur as unspecific side effects, i.e. symptoms not explainable by the drug, or may also exacerbate specific side effects [9]. Studies which investigated adverse events after placebo intake [10, 11], as well as studies which used verbal suggestions to manipulate treatment expectations prior to medication intake, demonstrated the existence of nocebo effects [12–14].

One of the main underlying mechanisms for the development of nocebo effects is expectations. These are formed based on prior experiences (either specific to the treatment or in general, to medical procedures) [15], social learning [16], and the information received about the treatment [17].

In clinical practice, patients are briefed about the treatment including potential risks in the informed consent. Although a great achievement towards patient autonomy, standard informed consent procedures may facilitate negative expectations which can, in turn, cause nocebo effects [18]. As reported in a meta-analysis, negative pre-treatment expectations predict the occurrence of actual side effects [19]. The strongest associations between symptom expectation and symptom occurrence were found for pain, fatigue, and nausea [19]. Moreover, negative treatment expectations prior to chemotherapy are high. In a study by Hofman and colleagues [20], most of the cancer patients expected that they would develop severe or long-lasting side effects such as fatigue (90.6%) and nausea (78%). In summary, optimizing informed consent procedures may be an essential module in the prevention of nocebo effects during chemotherapy.

By acknowledging the ethical and legal necessity of informed consent on the one side and the importance to minimize risks on the other, Barsky and colleagues [21] suggested educating patients about the nocebo effect. When aware of the existence of expectation-induced nocebo effects, patients’ risk of misattributing unspecific symptoms to the treatment may be lower and they may be less vigilant to bodily signals, which again, may decrease the number and intensity of symptoms [22]. First evidence has shown that informing participants about the nocebo effect led to a reduction of symptoms that were allegedly caused by wind turbines [23]. Despite the high relevance of preventing nocebo effects during routine chemotherapy treatments, no comparable research has yet been conducted in the clinical context.

The primary aim of this exploratory clinical trial is to investigate the effects of a nocebo-education on the patient-rated number and intensity of side effects in gastrointestinal cancer patients undergoing chemotherapy. By suggesting that side effects are not thoroughly determined by the pharmacodynamics of the treatment, patients’ perceived self-efficacy to control side effects may be increased, and the misattribution of ambiguous body signals to the treatment may be reduced. Additionally, the nocebo-education may also facilitate the development of positive expectations in general, which in turn, can improve health outcomes [24]. Thus, we will investigate, as secondary outcomes, patients’ perceived coping with side effects, tendency to misattribute symptoms, compliance intention, attitude towards the chemotherapy, clinician-rated severity of toxicity and type of co-medication used to treat side effects. Furthermore, we will examine whether optimized treatment expectations mediate the hypothesized beneficial effects of the nocebo-education. Since monitoring information coping styles have been associated with a higher report of side effects [25], we will investigate treatment information seeking as a moderator.

## Methods

### Study design

The RENNO trial (German Acronym for “**R**eduzierung des subjektiven **E**rlebens von **N**ebenwirkungen während der Chemotherapie durch Aufklärung über den **No**cebo-Effekt”) is a randomized-controlled, single-site superiority trial with two parallel arms and a primary endpoint of patient-reported number and intensity of side effects at 10 days and 12 weeks after the first chemotherapy session. Patients with newly diagnosed gastrointestinal cancers scheduled for chemotherapy are block-randomized 1:1 to a nocebo-education group or an attention control group entailing a quality-of-life interview.

The intervention takes place < 24 h before chemotherapy commences or during the administration of the first dose. Measurements are taken before (T1-pre), and after the intervention (T1-post), and 10 days (T2) and 12 weeks (T3) after the initial chemotherapy (Table 1).

**Table 1:** Schedule for enrolment, interventions and assessments

Time points	Study period				
	Allocation	Post-allocation			
	t_1pre_	t_1_	t_1post_	t_2_	t_3_
ENROLMENT					
Eligibility screen^a^	✓				
Informed consent	✓				
Randomized assignment	✓				
INTERVENTIONS					
Nocebo-education group		✓			
Attention control group		✓			
ASSESSMENTS					
Baseline variables					
Demographic and medical characteristics	✓				
Distress Thermometer	✓				
Information coping style	✓		✓		
Outcome variables					
Side effect number and intensity (GASE)^b^				✓	✓
Coping with side effects (GASE-Coping)				✓	✓
Misattribution of symptoms (GASE-Attribution)				✓	✓
Co-medication to treat side effects (yes/no)				✓	✓
Compliance intention	✓		✓	✓	✓
Attitude towards the chemotherapy	✓		✓	✓	✓
Severity of toxicity (CTC)^c^	✓			✓	✓
Further variables					
Expected side effects (GASE-Expect)	✓		✓		
Expected coping with side effects (GASE-Coping Expect)	✓		✓		
Expected efficacy of chemotherapy			✓		
Relevance of the conversation			✓		
Knowledge about the nocebo effect^d^			✓	✓	✓
Disease progression				✓	✓

*Note.* The time points T1-pre and T1-post refer to the intervention whereas the T2 and T3 refer to the chemotherapy (10 days and 12 weeks after the initial chemotherapy). *GASE* Generic Assessment of Side Effects, *CTC* Common Toxicity Criteria

^a^The eligibility screening takes place in two steps. In the first step, medical eligibility criteria are checked, whereas in the second step, all further criteria are assessed as part of the health professional-patient communication right before the signing of the informed consent

^b^Assessed symptoms include fatigue, nausea, diarrhea, vomiting, headache, shortness of breath, and rash

^c^Severity of toxicity is assessed by the clinician at every cycle

^d^Knowledge about the nocebo effect is only assesses in the nocebo education group

### Trial registration and ethical approval

Ethics approval has been given by the local ethics committee of the University of Hamburg. The trial has been retrospectively registered on March 27th, 2018 at the German Clinical Trial Register (ID: DRKS00009501).

### Study sample

Patients with newly diagnosed cancer of the gastrointestinal tract, including the esophagus, stomach, colon, rectum, anus, pancreas, gallbladder and bile duct who are indicated for chemotherapy are eligible for this study. Chemotherapy can be given in the adjuvant, neoadjuvant, or first-line palliative setting, and is administered as a mono chemotherapy or combination-chemotherapy, with or without vascular endothelial growth factor (VEGF)-antibody. To enroll, patients must be at least 18 years old, have no difficulties understanding German, and be able to give informed consent. Exclusion criteria include the following: any chemotherapy before trial inclusion, limited capability of self-care (Eastern Co-operative Oncology Group (ECOG) ≥ 3 [26]), severe comorbid psychological disorder (schizophrenia, substance abuse, severe depression or severe anxiety disorder), concurrent psychotherapeutic or psychopharmacological treatments, life-threatening comorbid medical condition, diagnosis of chronic skin disease, dyspnea, or chronic lung diseases due to its interference with chemotherapy side effects, indication for epidermal growth factor receptor (EGFR)-antibody treatment since it may cause acne-like rash. Since prior research has found that the link between negative treatment expectations and side effects was more pronounced among patients who had chemotherapy experience [19], we excluded patients with prior chemotherapy experience, including having received the first dose before enrolment, to achieve an increased homogeneity of the sample.

### Recruitment

Patients are recruited from the University Medical Center (UMC) Hamburg-Eppendorf, Department of Oncology, Hematology and Bone Marrow Transplantation and from cooperating practices. All patients are treated in an outpatient setting. With the aim to achieve adequate enrolment of participants, each patient with a newly diagnosed gastrointestinal cancer treated at the Department of Oncology of the UMC is screened. The first screening of inclusion criteria is conducted by the patient’s treating oncologist.

The healthcare professional (medical doctoral candidate or Psychologist, B.Sc.) informs patients about the study’s aim (“gaining an enhanced understanding of the quality-of-life of patients which undergo chemotherapy including potential worries and expectations about the treatment”) and procedures (“assignment to one of two possible conversations”). Further eligibility criteria are assessed through the healthcare professional by inquiring the patient directly (e.g. ECOG score). Informed consent is obtained by the same healthcare professional prior to enrolment.

### Randomization and blinding

A stratified block randomization is performed after the signing of the informed consent. To prevent a potential selection bias due to predictability, the block sizes are not noted here. The stratum distress is assessed at T1-pre using a distress-thermometer, a commonly used screening instrument among oncological patients ranging from 0 (not distressed) to 10 (extremely distressed) [27]. The allocation sequence for each stratum category (distress < 5 ‘low’ vs. distress ≥5 ‘high’) is produced by a research assistant prior enrolment using a computer program [28]. Sequentially numbered, opaque, sealed envelopes are used for the concealment of group assignment. Randomization is performed by a trained healthcare professional who, according to the distress level indicated by the patient, draws the envelope from the respective box. Patients are present during the allocation and are informed about their assignment instantaneously.

Due to the nature of the study, patients are not blinded. However, they are unaware of the specific study hypotheses and the content of the intervention that they do *not* receive. The trained healthcare professional is not blinded to group assignment. However, we aimed to confine the post-randomization interaction between the trained healthcare professional and the patient to the intervention. Follow-up assessments are conducted independently via mail using a standardized cover letter, i.e., no further interactions take place. Only in cases when patients do not complete assessment, the healthcare professional conducts a reminder call which is manualized in advance.

### Interventions

Both interventions take place right after the randomization and are carried out by trained healthcare professionals in an empathetic, patient-centered manner and are of similar duration (15–20 min). The attention control group controls for unspecific effects like attention by the healthcare professional or a positive patient-practitioner relationship. The intervention is conducted in addition to clinical routine and is scheduled individually between the healthcare professional and the patient to achieve the best fit in line with the patient’s schedule. Pre-defined as exclusion criteria, concomitant psychotherapy or psychopharmacological treatments are prohibited. Also as part of the clinical routine, all patients receive prescriptions for medications against nausea/vomiting and diarrhea.

#### Nocebo-education group

The nocebo-education is a manualized information session with the objective of illustrating the nocebo effect in a comprehensive way using patient-oriented language and practical examples [18]. It was developed as part of an experimental pilot-study by the research team of the PI of this study (YN) and has been adapted according to patient feedback. The intervention includes the following five steps: (1) The healthcare professional asks the patient about their personal experiences with side effects of medications or other treatments. (2) A case example of a nocebo response in the clinical context is introduced and discussed (adapted from [29]). (3) A standardized leaflet about the nocebo effect is handed out (see Table 2) and discussed. Patients are invited to talk about their own thoughts and potential prior experiences with nocebo responses. (4) Lastly, patients are encouraged to apply the obtained information to their imminent chemotherapeutic experience. The manual accounts for potential deviations of patients’ experiences and recall abilities (e.g. if the patient cannot recall any individual nocebo responses, the health professional may use a personal example).

**Table 2:** Brief discussion points from the leaflet of the nocebo-education

Information sheet about side effects	
A) The occurrence of side effects has two essential causes: • Pharmacological (substance-dependent) causes which activate biochemical reactions in the body • Psychological (non-substance dependent) causes which mainly include prior experiences and expectations of patient and relevant aspects of the therapeutic context (called the nocebo effect)	
B) Nocebo effects: education and exemplification • Nocebo effects are no illusions, but real and biologically measurable effects. • Prior negative experiences with adverse effects of drugs or even just reading about them in a medication leaflet can increase negative expectations of developing side effects. This might in turn lead to an increased actual occurrence of side effects.	
C) Case example of nocebo effects: • *“For my next checkup, I was to receive a contrast agent. I was anxious, knowing that my body reacts strongly to that kind of thing. The nurse hooked me up to the IV, through which the contrast agent would enter my body. She told me that the contrast agent would make me feel hot and that there might be a burning sensation. She then left me alone. The minute she left the room, I felt the heat washing over me, it streamed through my body and it burned. I knew this checkup was going to be awful. I felt extremely frightened. After a few minutes the doctor entered the room and she told me: Ok, let‘s inject the contrast agent, shall we?*” *(adapted from* [29]*)*	
D) Empathetic encouragement to explore own examples of nocebo effects. Gain reassurance that possible adverse effects of chemotherapy may be affected by own expectations.	

#### Attention control group

A face valid topic of matching relevance to the research topic of this trial is discussed with the patients in the attention control group in a similarly manualized manner. Based on the Functional Assessment of Cancer Therapy general scale (FACT-G; [30]), the healthcare professional makes inquiries about the quality-of-life subthemes physical well-being, emotional well-being and functional well-being using a semi-structured interview format. The two themes beliefs and spirituality, and relationship to practitioners which are not part of the scale are additionally addressed. Twenty questions are asked in total. The healthcare professional or the patient may address issues in further depth. Some items are rephrased to achieve a balance of negatively and positively phrased items (e.g. “I am worried that my condition will worsen” is rephrased as “I am optimistic about the future”). Answers are noted on the questionnaire. Answers to the interview are not part of the research questions and are hence not analyzed.

### Data collection and confidentiality

Self-report data is collected at before and after the intervention (T1-pre and T1-post) by the healthcare practitioner. Further self-report data at 10 days and 12 weeks follow-up is collected via mail. Medical characteristics are assessed using medical records. Clinician-rated data are collected at every chemotherapy cycle. All data in paper form will be stored at the UMC Hamburg-Eppendorf in rooms inaccessible to non-research staff. Since we conduct repeated measurements, identifying information of the patient is substituted by a code. Patients’ personal information (name, phone number, address) are securely stored separately from data which are outlined under “measures”.

### Measures

#### Primary outcomes

The mean group difference in number and intensity of patient-reported side effects at 10 days and 12 weeks after the initial chemotherapy session are defined as primary outcomes. Based on the Generic Assessment of Side Effects (GASE) [31], we chose four side effects specific to the chemotherapy, i.e., nausea, vomiting, diarrhea, and fatigue [32, 33]. Three unspecific symptoms, i.e., headache, shortness of breath, and rash, were defined as side effects that were very unlikely to occur in pharmacodynamic relation to the chemotherapy. They were chosen as a result of discussions among the research staff oncologists. A global item assesses the overall intensity of side effects in the last 7 days. The original GASE ranged from 0 to 3; to optimize outcome sensitivity in this exploratory trial, we increased the item range from 0 (*not at all*) to 10 (*very much*). Numbers of side effects are computed by adding up the number of present symptoms (≥ 1).

#### Secondary outcomes

Coping with side effects, the tendency to misattribute symptoms, compliance intention, attitude towards the chemotherapy, co-medication to treat side effects, and clinician-rated severity of toxicity measured at 10 days and 12 weeks after the initial chemotherapy session are examined as secondary outcomes. Coping with side effects is assessed as part of the GASE (GASE-Coping; varied after [34]). For each side effect, patients are asked to indicate how well they felt able to cope with the symptom (0 ‘*not at all*’ to 10 ‘*very much*’). Accordingly, the tendency to attribute symptoms (“To which degree do you attribute [symptom] to the chemotherapy?”) is assessed for each side effect on the same scale. The tendency to misattribute is computed as the mean attribution tendency of unspecific symptoms to the chemotherapy. Compliance intention to chemotherapy is assessed with two items (“How sure are you that you will complete your chemotherapy cycle?” from 0 “*not at all*” to 10 “*very much*”; “How high is the probability that you will discontinue your chemotherapy?” from 0 to 100%). Attitude towards the chemotherapy is measured using one item (“How would you describe your attitude towards the chemotherapy”) which ranges from 0 (*very negative*) to 10 (*very positive*). Co-medication to treat side effects is assessed with a yes/no question. The clinician-rated severity of toxicity is assessed using the Common Toxicity Criteria (CTC) Version 4.03 at every cycle. The CTC provides standardized definitions of adverse events ranging from grade 1 to grade 5 with each grade reflecting a distinct description of the adverse events severity [35].

#### Mediating variables

Expected side effect intensity and expected coping with side effects are assessed with a modified version of the GASE [34] before (T1-pre) and after the intervention (T1-post). Patients indicate the expected intensity and coping for each of the 7 symptoms (0 ‘*not at all*’ to 10 ‘*very much*’).

#### Baseline variables

Gender, age, education, employment, psychological distress [27], the affected gastrointestinal area, physical and psychological co-morbidities are assessed using self-report. Tumor staging, aim of treatment (neoadjuvant, adjuvant, palliative etc.), and the chemotherapeutic regime are obtained from medical records. Information coping styles are assessed with two items (“To which degree do you wish to be informed about the chemotherapy?”, “How strongly do you feel the need to inform yourself about potential side effects?”), both rated on a scale of 0 (*not at all*) to 10 (*very much*).

#### Manipulation check, evaluation of the nocebo-education, process variables

Comprehension of the nocebo effect information is reviewed by asking patients in the nocebo group to describe the nocebo effect. Perceived relevance of the nocebo-education and the control intervention is assessed at T1-post. Expected efficacy of chemotherapy includes four items that assess to which degree the patient anticipates the treatment will 1) help them recover, 2) shrink the tumor, 3) improve their quality-of-life and 4) prolong their life. Each item ranges from 0 (*not at all*) to 10 (*very much*). Progression of cancer is assessed from medical records during the study period.

### Sample size calculation

In a pilot study which aimed to validate the nocebo-education session, we found a moderate to large effect (*Cohen’s d* = 0.50–0.65) when comparing post-session side effect expectations between participants who were educated vs. those who were not educated. Since no prior study has been conducted to examine the effect of a nocebo education on side effects, we used the aforementioned effect size of side effect expectations as a proxy for actual side effects. Hence, when conducting an independent *t*-test, given an effect size of *d* = 0.60, the desired power of 80%, and an alpha-error of 0.05 (two-tailed testing), we obtained a required sample size of *N* = 45 per group for this exploratory clinical study. When accounting for a dropout rate of 10%, a total sample of *N* = 100 is needed to investigate potential group differences with adequate power for our primary outcome burden of side effects.

### Data management

After data entry, 10% of the electronic data is checked for correspondence with the original paper data. Plausibility checks (e.g. for item range and scale ranges) are conducted.

### Statistical methods

All outcome analyses will be conducted for the intention-to-treat sample. Missing values will be imputed using the expectation-maximization algorithm. We will conduct ANCOVA for the primary outcomes number and intensity of side effects, and for the secondary outcomes coping with side effects, tendency to misattribute symptoms, compliance intention, attitude towards chemotherapy, and medical toxicity at both time points (10 days and 12 weeks) with adjustments for the stratum distress and for cancer staging. Logistic regression will be conducted for the secondary outcome co-medication to treat side effects with the same set of covariates. The T1-pre to T1-post changes in expected side effects and expected coping with side effects will be examined as mediators of the effect of group on the outcomes. Exploratory analyses will be conducted to compare the expected efficacy of the chemotherapy between groups using independent t-tests.

Moderate analyses will be carried out by including the group x information coping style interaction in the ANCOVA. Sensitivity analyses will be conducted under exclusion of (1) patients of the nocebo-education group who either indicated to not have heard about the nocebo effect or who gave a wrong description and (2) patients with tumor progression.

No interim analyses will be conducted.

## Discussion

This is the first study to evaluate whether a nocebo-education can have beneficial effects considering side effects of the chemotherapy when compared to an attention control group. Up to now, no study has evaluated whether informing patients about the nocebo effect would be useful to optimize expectations and decrease side effect load in clinical practice. If efficacious, this trial would contribute to solving the ethical dilemma posed by the necessity and importance of informing the patients about the treatment on the one side and the potential negative effects of the information on the development of side effects on the other. Our objective was to create a nocebo-information session which is short, patient-oriented, and feasible in regard to implementation into clinical routine. Also, since the nocebo-education can be provided additional to the practitioner’s information, no further changes to the clinical practice are necessary.

The following limitations should be noted: As the first exploratory trial to test hypothesized effects of a nocebo-education, the sample size in this study is rather small. Also, no specific power calculation was possible since the effects of a nocebo-education on side effects are unknown. Given the link between side effect expectations and side effects, we based our power calculations on the effects of a nocebo-education on side effect expectations. Nonetheless, the use of a proxy outcome for the sample size calculation constitutes a limitation. Due to the nature of the study, blinding of patients and healthcare professional was not possible. Whereas the healthcare professional is the assessor at T1-pre and T1-post, outcome assessments at 10 days and 12 weeks after the initial chemotherapy are conducted via mail to lessen potential biases through the assessor. Since our patient population is expected to be highly burdened, we aim to keep the additional effort which may arise through questionnaire completion to a minimum. Hence, except for our primary outcomes, we use several one-item or two-item questionnaires which have not been previously validated. Lastly, our primary outcome is based on patient self-report. If this trial shows beneficial effects of the nocebo-education, further studies including physiological measures or clinician-rated measures as primary outcomes would be valuable.

Preventing side effects of chemotherapy is essential. For example, fatigue alone can occur for months during the treatment, and for 20–34% of patients, persists up to 5 years after treatment completion [36, 37], perennially affecting quality-of-life [20, 38]. Side effects of chemotherapy are also linked to increased anxiety, insecurity and a feeling of loss of control [39]. Hence, the development of innovative interventions to reduce side effects can be beneficial for the patient in the short and in the long run, as well as for public health systems.

## Abbreviations

ACG: Attention control group
CTC: Common Toxicity Criteria
ECOG: Eastern Cooperative Oncology Group
EGFR: Epidermal growth factor receptor
FACT-G: Functional Assessment of Cancer Therapy-General scale
GASE: Generic Assessment of Side Effects
RENNO: Reducing subjectively perceived side effects of the chemotherapy by educating about the nocebo effect [Reduzierung des subjektiven Erlebens von Nebenwirkungen während der Chemotherapie durch Aufklärung über den Nocebo-Effekt]
VEGF: Vascular endothelial cell growth factor
